# Abundance and Dynamics of Small Mammals in New Zealand: Sequential Invasions into an Island Ecosystem Like No Other

**DOI:** 10.3390/life13010156

**Published:** 2023-01-05

**Authors:** Carolyn King

**Affiliations:** Environmental Research Institute-Te Pūtahi Rangahau Taiao, School of Science, University of Waikato, Hamilton 3240, New Zealand; cmking@waikato.ac.nz

**Keywords:** invasive species, population irruptions, New Zealand

## Abstract

New Zealand had no people or four-footed mammals of any size until it was colonised by Polynesian voyagers and Pacific rats in c. 1280 AD. Between 1769 and 1920 AD, Europeans brought three more species of commensal rats and mice, and three predatory mustelids, plus rabbits, house cats hedgehogs and Australian brushtail possums. All have in turn invaded the whole country and many offshore islands in huge abundance, at least initially. Three species are now reduced to remnant populations, but the other eight remain widely distributed. They comprise an artificial but interacting and fully functional bottom-up predator-prey system, responding at all levels to interspecific competition, habitat quality and periodic resource pulsing.

## 1. Introduction

### 1.1. Historical Background

In most northern hemisphere countries, small mammal communities develop over evolutionary time, according to semi-predictable assembly rules. The structure of a local community is determined by the interactions among the resident species past and present, modified by geography, resource distribution, climate, immigration opportunities, mutual interactions and long evolutionary history. Analyses of existing models of community structure employ a great range of functional parameters and varieties of species composition. They all assume assembly rules that are entirely natural, the result of long-term processes at continental scale, including dispersal, resource partitioning, predation and competition, plus historical inertia [1]. Only in New Zealand are the living communities of small land mammals entirely un-natural, and yet still functional.

New Zealand comprises an archipelago of islands in the southwest Pacific Ocean (34–46° S), about the same size as the British Isles but generally warmer because it is closer to the Equator than is Britain (50–58° N). It is the only emergent section of Zealandia, a segment of continental crust the size of India. Zealandia separated from the edge of Gondwanaland during the late Cretaceous period. Continental fauna and flora typical of that era were carried eastwards into isolation, gradually losing ancient species by extinction and acquiring new ones by immigration [2]. Over time, Zealandia was stretched thin and steadily lost buoyancy, so by the Oligocene era, almost all of that fragment of continental crust was under water [3]. A handful of its former residents remained (including primitive frogs, lizards, snails and invertebrates) if they were small enough to survive on the few scattered islands still dry [4]. The only land mammal among them (that we know of) was a small extinct representative of the Miocene stage of mammalian evolution, pre-dating the earliest division between the placentals and the marsupials [5].

By the late Tertiary era, earth movements and volcanism were reversing the sinking process. A reinvigorated landmass emerged, down-wind and down-current from Australia, and was rapidly colonised by animals and plants that could cross the intervening water barrier unaided. Birds, insects and bats flew across what is now the Tasman Sea in their thousands, and a huge variety of marine vertebrates (whales, dolphins, seals, sea-lions, penguins, and a vast array of sea-birds) thronged the surrounding waters. They were isolated from the rest of the world by open oceans stretching >2000 km in every direction. No strictly land mammals could make such a trip.

Back in Gondwanaland, ongoing evolution produced a great variety of four-legged land mammals and venomous snakes, but these (among many others) could neither swim nor fly far enough to cross the widening Tasman Sea. Meanwhile, the isolated archipelago now known as New Zealand supported the evolution of a complete functional community of endemic species, dominated by birds, bats and reptiles. They adapted to fill almost all the niches normally occupied by land mammals, and spread across the 27 million ha of emergent land available, most covered in forest from sea level to alpine heights [2]. This extraordinary history made the islands and their inhabitants an environment like no other on the planet.

All the four-footed small mammals now present in New Zealand have arrived with human help, both accidental and deliberate, in a sequence determined at first by eighteenth-century historical exploration and trading links, and later by nineteenth-century errors of judgement. Key small mammal species native to the northern hemisphere (voles, lemmings, shrews, moles, dormice, woodmice, gophers, and squirrels) remain absent. Over time, the introduced rodents, lagomorphs and their predators have each in turn adapted to an environment totally different from that of their ancestors, often replacing the earlier residents in a process aptly labelled ‘Feathers to Fur’ [6].

### 1.2. The Arrival of People

The first Polynesian voyagers, sailing southwest from the Society Islands or nearby groups, discovered the land they called Aotearoa in about 1280 AD. They were accompanied by their domestic dogs, and by the Pacific rat—the very first rodent to run wild on land that had not known any such animal for millenia past. The only native small mammals they found were bats, plus seasonal assemblages of pinnipeds hauled out on their breeding rookeries round the coasts. Much later, in 1769, the rest of the world broke in after the first visit of the English explorer James Cook, followed in due course by waves of European whalers, sealers, traders and settlers, with at least 51 mammalian companions [7].

Eleven species of introduced small mammals are considered here: four commensal rats and mice, three mustelids (weasel, stoat and ferret), one insectivore (hedgehog), one lagomorph (European rabbit), one felid (domestic cat) and one marsupial (Australian brushtail possum). All four rodent species and the cat were introduced accidentally, because they could not be prevented from landing along with their human companions. All three mustelids, the rabbit, the hedgehog and the possum were introduced deliberately, for reasons that seemed good at the time.

This paper is a review and synthesis of the abundance, dynamics and interactions of the small mammalian species and their predators caught up in this un natural historical experiment, summarized in Table 1 and in greater detail elsewhere [8]. 

**Figure 1:**
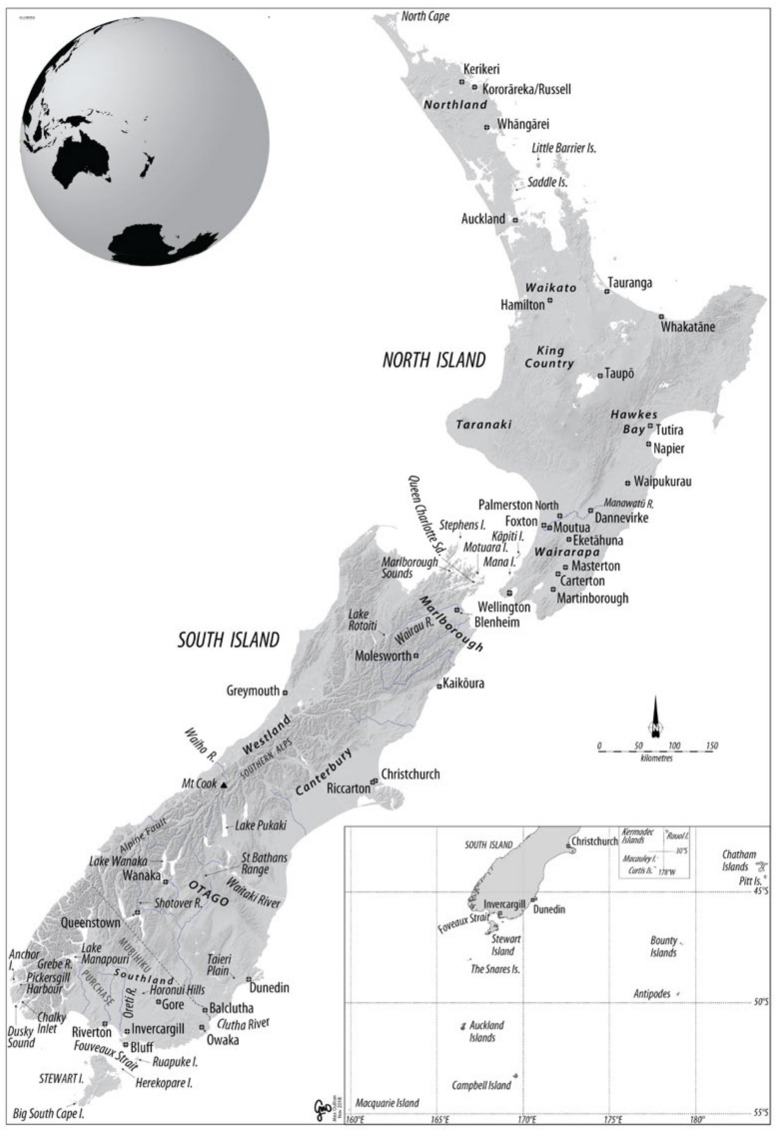
Map of New Zealand, with place names mentioned in the text. Cartography by Max Oulton.

**Figure 2:**
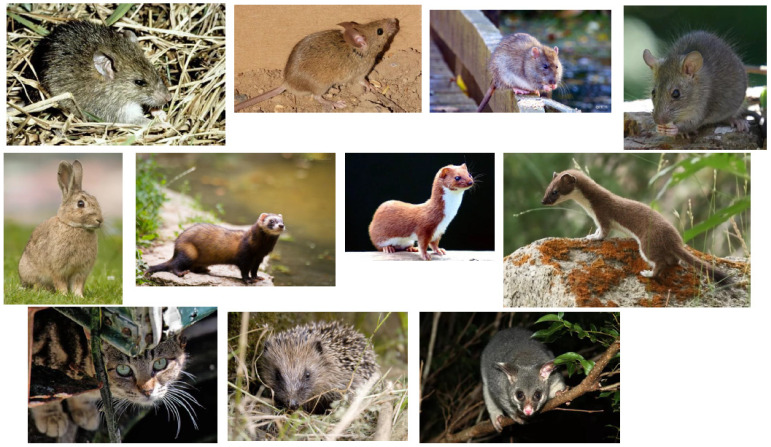
Eleven introduced small mammal species in New Zealand. Top row, left to right: *Rattus exulans, Mus musculus, R. norvegicus, R. rattus.* Middle row: *Orycolagus cuniculus, Mustela furo, M. nivalis, M. erminea.* Bottom row: *Felis catus, Erinaceus europaeus, Trichosurus vulpecula*. For copyright attributions, see Acknowledgements.

The red fox *Vulpes vulpes* was declared a prohibited import from the earliest days of European colonisation. Here Figure 1 and Figure 2.

## 2. Pacific Rat, *Rattus exulans* (Peale, 1848)

The Pacific rat is the smallest of the three species of rats in New Zealand. Adult males seldom exceed 100 g in body weight, and females slightly less, but their age and size varies widely between populations, and between years.

*R. exulans* is widely distributed across mainland southeast Asia and on most Pacific islands. In around 1280 AD these little rodents accompanied the last wave of prehistoric human migration from the Society Islands region to New Zealand. Their genetic signatures excavated from archaeological sites around the Polynesian islands confirm their travels, and offer important proxy evidence for exploring otherwise undocumented human dispersal patterns [9].

### 2.1. Arrival

The arrival of Pacific rats is firmly dated from many clear signals of their earliest presence in a previously rodent-free environment, all entirely consistent with other palaeoecological evidence. The sudden intrusion of people and their rats and dogs into a world previously dominated by birds and reptiles had an immediate, catastrophic and still detectable effect on New Zealand’s ecology from the late 13th century onwards. Radiocarbon dating of the first seeds to show rodent toothmarks [10], and of damage to shells of giant landsnails (*Placosytylus ambagiosus*) [11]; the start of widespread deforestation [12], of extensive megafaunal extinctions [13], and of a pre-European decline of marine mammal populations [14], all confirm that Pacific rats and their human companions arrived in New Zealand at about the same time.

### 2.2. Abundance

The first Pacific rats found a novel ecosystem previously free of any mammalian predators, in which food resources, from rich vegetation to ground-feeding birds and bats to lizards and giant invertebrates, were enormously abundant. They must have reached great numbers, and were certainly once very widespread. Their bones have been found in coastal middens, sand dunes, rock shelters, and limestone caves throughout the North, South, Stewart, Chatham and Great Barrier Islands, and on at least 40 smaller offshore islands ≥5 ha.

Since 1984, the New Zealand Department of Conservation (DOC) has eradicated rats of all species from as many offshore islands as possible. The only large island on which the population dynamics of Pacific rats had previously been studied was Tiritiri Matangi (188 ha), a de-forested (cleared for farming) grassy island which was monitored for six successive years before the rats were eradicated in 1993. No other rodents or mustelids were present, and cats had been eradicated in the 1970s. A short, intense breeding season produced a regular annual birth pulse, larger when the annual crop of grass seed was good [15]. In the years 1975 to 1980, Pacific rats reached annual densities of 100–170/ha in grassland, and 70–80/ha in the few remnant forest patches, varying strongly with season, and between years and habitats.

The abundance of all rodents is strongly affected by food supplies, correlated with vegetation cycles. On the main islands, forests dominated by the southern beeches (Family Nothofagaceae) and native softwoods such as rimu (Family Podocarpaceae), among others, typically produce huge crops of seed every few years (known as ‘mast years’). The huge pulse of food resources suddenly available during a masting event has a cascading effect on the resident biota, including a predictable population irruption of forest rodents and seed-eating birds. Māori hunted Pacific rats for food, so of course they were well aware of such temporary bonanzas [16]. In the late 19th century, irruptions in the northern South Island were reported in the European literature in 1872, 1878 1880, 1884 and 1888 [17], and this is certainly an incomplete record. The dynamics of these food driven irruptions parallel those observed today in mice [18,19].

### 2.3. Predators and Competitors

After 600 years of being the only resident rodent throughout New Zealand, Pacific rats suddenly disappeared from most of the North Island during 1850–60, and from most of the South Island in the 1890s. Their retreat coincides with the arrival and rapid spread (over <80 years) of house mice (*Mus musculus*), and of Norway rats (*R. norvegicus*) disembarking repeatedly from European ships. The last mainland Pacific rats survive as a few remnant populations in Fiordland, and on islands as small as 5 ha, but their distribution on most islands (especially small ones) has been reduced since 1984 by eradication operations.

Competition with (or predation by) European rats may certainly have contributed to the rapid retraction in historic range of Pacific rats, but identifying the process is not easy. Early European observers, from Darwin to Dieffenbach [20,21], later echoed by Wodzicki [22] and Watson [23], attributed the decline and disappearance of Pacific rats to the spread of Norway rats. On the other hand, all three rat species co-exist around the Pacific, and on Stewart Island, where mice (and stoats) are absent. Recent computer modelling suggests that the absence of Pacific rats on New Zealand islands is most strongly linked with the presence of ship rats, and less so with that of Norway rats [24].

Native predators of Pacific rats capable of taking mice and young rats include a small forest owl (*Ninox novaeseelandiae*), a kingfisher (*Halcyon sancta*) and the weka, an aggressive ground-feeding member of the Rallidae (*Gallirallus australis*). The Australasian harrier (*Circus approximans*) used to visit Tiritiri Matangi Island in autumn to hunt Pacific rats when they were abundant. Bones of Pacific rats are often found concentrated in the roosting sites of the now extinct laughing owl (*Sceloglaux albifacies*) [25].

Pacific rats were once the staple diet of cats on Little Barrier Island and on Raoul Island, but both islands are now free of both rats and cats. The larger rats are certainly capable of killing and eating mice [26] and Pacific rats. Mustelids arrived too late to meet any Pacific rats on the mainland except for a few remnant locations in the southern the South Island, and they are absent from nearly all the islands still inhabited by Pacific rats.

By far the most significant predators of Pacific rats are human conservation authorities. Pacific rats are reckoned to have contributed to the extinction of at least 23 bird species in New Zealand [27]. New Zealand biodiversity had been exposed to predation by Pacific rats for at least half a millennium before any other mammalian predators arrived. The native species most at risk were lizards, invertebrates and birds that were small (<200 g bodyweight) and flightless and/or had small eggs [28]. To protect what endemic biota remains, eradication operations have become routine, cost-efficient and increasingly successful at larger and larger scales [29]. Pacific rats have gone from at least 38 offshore islands larger than 5 ha, and 20 smaller ones.

On the other hand, the DOC also recognizes that Pacific rats (in Māori, *kiore*, pronounced *kior-eh*) are taonga (treasures) for some Māori, and in 2010 the Ngatiwai Trust Board and DOC agreed to provide a refuge and protective status for kiore on Mauitaha and Araara islands in the Hen and Chickens group. Elsewhere, the story of the rise and fall of Pacific rats in New Zealand is now complete.

## 3. House Mouse, *Mus musculus* Linnaeus, 1758

*Mus musculus* is a single polytypic species, *Mus musculus* Linnaeus 1758, with at least three recognisable subspecies with separate geographic ranges. New Zealand is one of very few places in the world where all three subspecies meet: *M. m. castaneus* (Waterhouse, 1843), native to Sri Lanka to South East Asia; *M. m. musculus* (L.), from Eastern Europe to Japan, across Russia and northern China; and *M. m. domesticus* Schwarz and Schwarz 1943, from western Europe and the Mediterranean basin. None of the subspecies is reproductively isolated from the others, so they are capable of genetic exchange, although not all hybrids are equally fertile. New Zealand hosts a complex mix of the three subspecies, dominated by *domesticus*, but also including many traces of *castanaeus* and a few of *musculus* [30]. Thanks to a decade-long collaboration by a team of students, historians and geneticists, the story of the invasion of New Zealand by house mice is the best known for any other island in the world [31].

Average body weights range between 15 and 25 g, slightly more in males than females, but mice continue to grow throughout life, so body measurements have to be corrected for location, age, season, and year.

### 3.1. Arrival

There have never been any native mice among the Pacific islands, so the first mice to reach New Zealand arrived as stowaways on American and European ships, and were carried ashore among food supplies and trading goods. Their origins and travel routes have been deduced both from documentary records and from shipping data [32]. The northern and southern ends of New Zealand received multiple invasions at different times and by mice of different ancestry, which have made their collective histories very complicated [31].

The southern ocean’s first unregulated commercial resources were the enormous breeding rookeries of fur seals and sea lions, yielding a rich but seasonal and unsustainable harvest of fur seal skins and seal oil lasting only from the 1790s to about 1820. The best fur market was in Canton, but trading New Zealand produce there was prohibited by the East India Company’s monopoly. The first mice to arrive in New Zealand were carried direct to the far south of the South Island, mostly Fiordland or the southern Otago coast, by early sealers willing to risk illegally selling sealskins in Canton. To avoid scrutiny in Sydney, they returned directly south on ships resupplied with Asian provisions, and Asian strains of both mice and Norway rats inadvertently travelled with them [30,33].

Hence, living mice in the southern South Island carry a history of incomplete hybridisation between Asian (*castaneus*) and European (*domesticus*) mice. These ‘semi-hybrid’ mice first occupied the whole of Southland, then spread northwards to a still-detectable contact zone, where they met pure-bred European *domesticus* moving south from Canterbury [34]. In the northern South Island, the Nelson area was settled independently by colonists from central Europe, bringing with them *domesticus* mice of German ancestry found nowhere else in New Zealand.

In the North Island, most mice are pure-bred *domesticus* from north-west Europe that arrived after the 1820s via Sydney (the first trading hub of the southwest Pacific, established in 1788). The many other *domesticus* lineages now living around the North Island are evidence of multiple later invasions of mice of different origins arriving throughout the 19th century. All six clades of *domesticus* are represented in New Zealand, most commonly E and F from Britain and Ireland, but at least a few mice from the other four clades are present, most originating from continental Europe. Once unloaded at ports around the country, mice were carried inland among stores for farms, sheep stations and settlements. Non-commensal house mice are now widespread throughout the North and South Islands, and on many offshore islands, but, notably, not on Stewart Island.

### 3.2. Abundance

The house mouse is usually thought of as typical of urban habitats, and of course it still is, but in New Zealand it is also widespread in native and exotic temperate forests, pasture, croplands and alpine areas from the coast to high altitude [35]. Because New Zealand never had any native mice, the house mice brought here quickly spread into a variety of non-urban habitats from which in Europe they are excluded by native species.

Population age structure varies with numbers, so far as can be told from broad age categories based on toothwear [36]. Juveniles usually enter the trappable population in summer and autumn, sometimes in large numbers. Mouse populations normally tend to peak in summer and autumn, declining through winter.

The length of the breeding season is determined primarily by the nutritional condition of the females, and their breeding rate is highest in older females, and early in the breeding season [19,37]. Mice usually stop breeding as the weather cools towards winter, but not after a masting event [38]. Massive proliferations of invertebrates and seeds on the forest floor [39] guarantee extended breeding through the winter [40]. Post-masting changes in mouse reproduction and high juvenile recruitment are reliably predictable from masting data alone [41].

Spectacular increases in mouse abundance indices in autumn and winter always follow a heavy beech mast in the austral autumn between March and May [18,42]. Mouse populations reach peak indices (captures per 100 trapnights) over the next few months, often around 25 C/100TN, up to (rarely) 77 C/100TN, and declining equally rapidly as recruitment fails from summer onwards. This short (12 month) cycle has predictable consequences for stoats and rats living in the same forests [43].

Capture rate indices can rise from zero to outbreak levels in a few months, due to changes both in numbers and in trappability, in unknown and variable proportions. Live-trap mark–recapture studies can confirm the remarkable population irruptions and crashes of wild house mice. Absolute density estimates of 27–50 mice/ha in late winter and spring (August and November) were recorded after a heavy beech seedfall in the Eglinton Valley in 1999, declining sharply over the summer to a minimum in February. By August 2001, in the absence of any useful beech seedfall in the 2001–03 seasons, <1 mouse/ha remained for the next two years [40]. Similarly, in forests dominated by rimu, a native podocarp (*Dacrydium cupressinum*), mouse abundance is positively correlated with the rimu nut crop, which in turn is determined by the fertility of the site. After a heavy rimu nut fall in Waitutu Forest in 2002, mouse density increased from <1/ha in August to 40–60/ha by February 2003 on each of six live-trapping grids, although only to <20/ha on three grids set on less fertile sites [44].

Above treeline, the normal summer density of mice varies seasonally up to 4.0 mice/ha, but a heavy flowering of the native snow-tussocks (*Chionochola* spp.) in the Borland Valley was followed by a spring density of mice peaking at 38.6 mice/ha in November 2006 [38].

### 3.3. Predators and Competitors

Mice greatly benefit wherever the other rodents are absent. Within fenced sanctuaries inaccessible to rats, mice can reach huge numbers, with damaging consequences for native invertebrates [45]. In Tawharanui Open Sanctuary, mouse density has reached 156.7 mice/ha in autumn, dropping to 14.6 mice/ha in mid-winter); on Antipodes Island, a *Mus*-only island before eradication (104/ha in dense coastal tussock). On the main islands outside a masting event, mice can coexist at least patchily with rats, probably by local habitat partitioning.

Ship rats actively hunt and kill mice [46], so mice survive best under thick ground cover where rats are scarcest [47]. Rats intimidate mice and discourage their foraging in open-floored forest, keeping local density indices for mice lower than in adjacent rat-free locations even where mouse productivity is the same in both [47]. That explains the clear reciprocal relationship between abundance indices for mice and rats in forests [26,48].

Stoats do kill many mice, but normally have no effect on their abundance. During and after a masting event, the functional and numerical responses of stoats to increased numbers of mice are out of phase. Stoats cannot remove mice fast enough [49] to prevent a post-masting irruption, because it begins over winter while stoats are still scarce. Stoats do stage massive functional and numerical responses to the increase in mice after a seedfall in autumn (March-May), but by the time an extra-large large cohort of young stoats appears six months later in early summer (November-January), mouse numbers have peaked, and are already beginning to decline [50]. Predation by stoats does not affect mouse numbers until after mouse populations have ceased to recruit young during the crash phase of the beech mast cycle [41,51]. The following decline in mouse numbers is inevitable, whether stoats are present or not.

Weasels are specialist hunters of small mammals, and they will respond to any potential opportunity to concentrate on mice, and are small enough to gain access to fenced sanctuaries where mice can become very abundant in the absence of rats. For example, 14 weasels have been trapped inside Maungatautari Ecological Island within 4 years [52]. However, the absence of voles normally keeps weasels relatively uncommon in New Zealand [53].

Predatory native birds take mice given the opportunity (moreporks *Ninox novaeseelandiae*, kingfishers *Halycon sancta*, falcons *Falco novaeseelandiae*, little owls *Athene noctua,* Australasian harriers *Circus approximans,* and weka *Gallirallus australis)*, but seldom in numbers sufficient to affect mouse populations.

Mice have certainly contributed to the historical decline in New Zealand’s biodiversity, especially the small vertebrates (lizards and frogs) and the invertebrates, but there are few precise data on the pre-European invertebrate fauna for comparison. Given the chance, mice are known to reduce the diversity and numbers of invertebrates—especially beetles, spiders, caterpillars, wētā and earthworms—which recover after mice are removed. On Mana Island, mice killed many McGregor’s skink (*Cyclodina macgregori*) and gecko (*Hoplodactylus maculatus*), but after the mice were eradicated in 1989, the numbers of both recovered [54]. In beech forests, mice support population irruptions of stoats that, in turn, are predators of native birds such as mohua (*Mohoua ochrocephala*), kākā (*Nestor meridionalis*) and kakariki (*Cyanoramphus auriceps*) [55].

## 4. Norway Rat, *Rattus norvegicus* (Berkenhout, 1769)

The Norway rat is the largest of the three *Rattus* species in New Zealand. Most wild adults weigh 150–300 g, but they can reach >500 g around rubbish tips and on offshore islands.

### 4.1. Arrival

Norway rats were the first European rodents to become established in New Zealand, scrambling ashore in the late 18th century from European and/or North American sailing ships [56]. In 1773 Cook observed them leaving the *Resolution* [57], and sealers and whalers frequently landed men, supplies and rats around the New Zealand coasts from 1792 to at least 1840.

The North and South Is are occupied by Norway rats of two unique and different genetic lineages, Norhap 01 and Norhap 2 [58]. Their ancestors arrived in New Zealand as two independent invasions, by different routes and from different sources. Those in the North Island, labelled Norhap 01, are descended from European rats arriving with traders and immigrants via Sydney. Those in the South Island, Norhap02, are descended from Asian rats that arrived with sealers direct from eastern ports, along with Asian mice. To avoid unwelcome regulations and taxes, the early sealers returning to New Zealand by-passed Sydney, which perhaps explains why no Norhap02 (or house mice with Asian ancestry) have yet been found there [33].

Kapiti, Chatham, Stewart, and Campbell Islands were all bases for large sealing and whaling fleets, whose rat-infested operations guaranteed early landing by ship-borne rats. The substantial volume of shipping that visited the Bay of Islands from the early nineteenth century onwards [59] distributed rats to at least eight islands in the Northland area. Ships still harbour Norway rats today, and remain a continuing source of new invasions of islands of conservation value [60].

### 4.2. Abundance

By the 1830s, Norway rats were ubiquitous in towns and bush throughout mainland New Zealand [61]. They lived in all types of habitat from coastline to mountain top, including above the snowline in the Southern Alps [62], and were seen in huge numbers on the beaches of Stewart Island However, by the end of the 19th century, early explorers were noticing a drastic change in the previous abundance of Norway rats. For no obvious reason, the formerly huge populations were fast disappearing from the main islands [63]. The largest numbers now remaining are commensal in virtually all towns and cities, and around farms and on cropland, especially where food and cover are always available (e.g., at rubbish tips).

Abundance estimated from trapping indices are indirect and potentially biased, because standard rodent snap traps are too weak to hold large rats, or miss them altogether, whereas steel jaw traps set for stoats in the same areas are more reliable [64]. Abundance estimates can reach very high figures on seabird islands occupied only by Norway rats. On Breaksea Island in 1988, at least 13 rats/ha were removed by a three-week intensive ground operation [65]. On Campbell Island in 2001, the Norway rat population had reached an abundance index of 123.5 captures per 100 trapnights before it was removed by a four-week baiting campaign [66]. Both islands are now staging a massive recovery of native fauna.

### 4.3. Predators and Competitors

All three rat species present in New Zealand have occupied most of the country at some stage, and all can have wide distributions in the absence of competitors, but all are now more or less restricted in the presence of any one of the others. More than one can coexist only on islands of >100 ha, but even then they partition strongly by habitat [24]. Norway rats arrived in New Zealand sooner than ship rats, and are much better swimmers, so Norway rats are, or once were, the more common of the two on offshore islands.

By contrast, the sudden disappearance of the once-abundant Norway rats from nearly all mainland forests in the late 19th century is now attributed to the spread of ship rats [67]. Superior climbing agility and efficient exploitation of arboreal resources by ship rats probably enabled them to out-compete Norways in forest habitats [68], typically confining Norways to foraging on the ground in commensal and riparian habitats [69]. Unfortunately, the arrival of ship rats coincided with that of stoats, so the effects of predation and competition on the decline of Norway rats on the mainland are now indistinguishable.

Cats and stoats can readily kill Norway rats, which in turn are known to kill house mice and Pacific rats. Norway rats disappeared from Resolution Island when stoats arrived. Conversely, Pacific rats benefitted when Norway rats were removed from Raoul Island (2965 ha), at least until they were also eradicated [70]. Pacific and Norway rats are seldom found together on smaller islands.

When Norway rats first arrived in New Zealand, there was still a great abundance of native fauna that lived, roosted or nested on or near the ground, especially frogs, reptiles, wetland birds and seabirds. Many of these species had survived, at least in reduced numbers, the depredations of Pacific rats during the Polynesian period, but were now further devastated by the newly invading Norway rats. This top-down impact of Norway rats on native biodiversity has had flow-on indirect effects to the entire trophic architecture and ecosystems of islands [71]. Hence, Norway rats have long been prime targets for restoration programmes. Of the more than 60 offshore islands known to have been colonized by Norway rats, they have so far been eradicated from about two-thirds of them, of which the largest was Campbell Island (10,891 ha) [72].

## 5. European Rabbit, *Oryctolagus cuniculus cuniculus* (Linnaeus, 1758)

### 5.1. Arrival

In early explorer days, domestic rabbits were carried alive as sources of fresh meat on ships, and were important items of trade and exchange (two pairs were gifted to local people by James Cook in 1777). Domestic rabbits were kept on the mainland and on offshore islands from the 1820s onwards. They are efficient small herbivores, but in the absence of grassy farmland, their preferred habitat, they did not then spread far.

Wild rabbits were first imported and released in Southland and Kaikoura in the late 1850s [73]. They followed the advance of sheep grazing inland across the dry, hitherto empty tussock lands of the eastern South Island. They spread with astonishing speed, until all suitable open country in the South Island (150,437 km^2^) was occupied by 1900. The wetter, mainly forested North Island (113,729 km^2^) took longer to convert to pasture. Rabbits spread more slowly there, not reaching most suitable habitat until 1948 [74]. Carcase weights of both sexes vary around 1.3 to 1.5 kg.

### 5.2. Abundance

By 1868, Southland farms began to report rabbit damage to crops [75]. On one property on the Oreti River, where the first rabbit was seen only in 1872, 26,000 rabbits were killed in a few months in 1876 [74]. From 1878, massive losses in lamb production and wool clip made huge areas of formerly profitable grazing lands uneconomic, and were abandoned as pastoralists gave up struggling against rabbit damage. Rabbits reached peak numbers in Otago in the 1880s, and extended onto less favourable habitats never occupied since.

The legendary damage caused by rabbits crippled pastoral farming in many areas during the 19th and early 20th century. Burning, burrowing, heavy cropping and trampling by sheep ruined native grasslands that had evolved without grazing mammals, and then the addition of many introduced weeds, changed the composition of the vegetation [76]. The resulting dramatically modified landscape suited rabbits well, and the absence of enough native natural enemies encouraged rabbit population explosions.

One early option to counteract declining wool yields was to ‘farm’ the rabbits. Exports of skins (earning 149.5 million pounds sterling during the 1920s alone) plus canned and frozen meat, continued until the rabbit was ‘decommercialised’ in the mid-1950s [77]. Rabbits still damage agricultural production, at the rate of NZ$1.1 to $2.1 per rabbit per stock unit, according to an early model assuming a gross margin per stock unit of NZ$21 [78], but nothing like as badly as they did at first.

Depending on location and feed availability, does may become pregnant at any time of the year, most commonly from June to March. Where New Zealand’s temperate climate and vast improved pastures (enriched with European grass species) allow a long plant growing season, rabbits can breed almost year-round. Annual productivity can reach 42–48 young per adult doe. In drier, colder areas, where winter conditions are tougher, the breeding season is short and sharply defined from spring to mid-summer (September to January). Litter size is no smaller (five or six), but only about 23 young per adult doe can be produced during the short breeding season. Kits born in autumn and early winter (February to May) seldom survive to become adults.

Rabbit abundance is very hard to estimate accurately, and certainly varies across a very wide range with location and season. Techniques for converting standard spotlight counts to absolute density remain controversial [79]. The relationship between spotlight counts and density is not linear, but when applied along fixed transects across 10- to 20 km units, well-organised counts can index relative changes over time [80]. In general, rabbit densities are low to moderate where rainfall is high and ground conditions permitting long wet vegetation are unfavourable for rabbits, hence their numbers seldom cause trouble. Dangerously high densities are now found only in semi-arid country where rabbits can increase to carrying capacity if left uncontrolled.

### 5.3. Predators and Competitors

The first rabbits to arrive in New Zealand met two native resident predators, the harrier hawk (*Circus approximans*) and the weka (*Gallirallus australis*), which both killed many rabbits, but not enough to slow the rabbit invasion. There were no native carnivores, and feral cats were not common until much later.

When rabbits became a serious pastoral pest, mustelids were imported and/or bred for release, and farmers bought up cats from towns to release on pastures teeming with rabbits. Ferrets and feral cats still dominate the suite of ‘natural enemies’ that hunt rabbits [81], but cannot control their numbers as well as the afflicted pastoralists believed they would. Feral cats certainly kill both adult and young above ground, and the elongated shape of mustelids enables easy hunting in rabbit burrows and breeding stops. However, contrary to popular belief, rabbits determine the numbers, habitat and diet composition of ferrets, stoats and feral cats, not vice versa [82].

On the other hand, the population ecology and abundance of rabbits can be understood only with reference to predator-rabbit relationships that typically range across a spectrum determined by habitat.

Where the breeding season is long, as in the warm and wetter parts of the North Island, a near-continuous supply of young rabbits maintains predator numbers throughout the year. Intense searching finds most rabbits even when they are scarce, so juvenile survival is poor [83]; in turn, predation keeps rabbit populations down to more or less low and stable levels.

Where the breeding season is short, as on cool and semi-arid parts of the South Island, the brief annual crop of young rabbits cannot maintain stable predator numbers. Predators leave when rabbits stop breeding in autumn, so then the higher survival of predator-free juveniles enables rabbit numbers to fluctuate widely with climatic conditions.

Predators do not regulate rabbit abundance nearly as effectively as do variations in climate, food, disease and habitat, unless rabbit numbers are low for some other reason (drought, poisoning or epidemics), after which predation can prevent rabbit recovery. Conversely, the numbers of ferrets and feral cats predictably decline or disperse following a successful rabbit control operation [84]. Some die after scavenging poisoned rabbits [85], and many take more native prey [86].

In all habitat types, rabbit numbers are most sensitive to juvenile mortality. In northern Canterbury, the mortality rate of nestling rabbits was higher where predators were present (51% of 71 litters), than where they were removed (32% of 55 litters). Post-emergent mortality (to 4 months of age) of radio-collared juvenile rabbits was high whether predators were removed (82% of 17 rabbits) or not (100% of 20). Few nestling young died from causes other than predation. On average only about ~40% of rabbits more than 6 months old survived another year [87].

New Zealand is one of the few countries with European rabbits but no myxomatosis. Proposals to introduce its specific carrier flea, *Spilopsyllus cuniculi* (lost during the long sea voyage of the colonising stock), followed by the myxoma virus from Australia, were withdrawn after an adverse environmental impact report [88]. Another rabbit disease, RHD, introduced illegally in 1997 by farmers deliberately spreading the virus smuggled in from Australia, reduced rabbit abundance considerably in most parts of New Zealand [89]. RHD has persisted in almost all areas where rabbits are present, causing periodic epidemics. The wider ecological effects of declines in rabbit abundance include increases in pasture biomass, increases in numbers of possums and hares, declines in numbers of rabbit-specialist predators, and short-term increases in predation rates of some native birds [90].

## 6. Ship Rat, *Rattus rattus* (Linnaeus, 1758)

### 6.1. Arrival

The ship rat was the fourth (and last) of the four commensal rodent species to reach New Zealand. Ship rats arrived in around 1830–50, when they replaced Norway rats as the commonest rat species aboard ships [91]. Ship rats are larger than Pacific rats but smaller than Norway rats, and as with all rats, the age and size of adults varies from year to year, with location, habitat, season, age; samples are biased by trap-type [64]. Males are heavier and longer than females, but in general the body weight of both sexes ranges between 100 g and 200 g.

Ship rats come in three freely interbreeding colour morphs: (A) ‘rattus’, with black backs and grey bellies; (B) ‘alexandrinus’, grey all over; and (C) ‘frugivorus’, with grey backs and white bellies. In any given place the dominant colour morph still demonstrates partial founder effects linked to the original colonists reaching that location [58].

Ship rats spread across the North Island from about 1860, and the South Island from about 1890 [24]. They quickly replaced the Norway rats that were until then still abundant everywhere, and finished off the last few Pacific rats. Ship rats are lightweight, agile climbers easily capable of accessing food above the ground, out of reach of the heavier and more aggressive Norway rats. The mild climate of lowland New Zealand forests, and the absence there of specialist, arboreal rodents such as squirrels, enables ship rats to live in the canopy all year round [68]. By contrast, ship rats in continental Europe are mainly commensal.

### 6.2. Abundance

Ship rats can live in almost any natural habitat and in the rafters and ceilings of buildings, in forests, towns, cities and farms on the mainland and on many offshore islands, but are most abundant in mature, diverse, lowland podocarp–broadleaf forests from the coast to the tree line, unaffected by partial logging [92]. In most years they are scarce in pure forests of southern beech (*Nothofagaceae*), and are normally rare at higher elevations [93], but will venture into the alpine tussock after a masting event [94]. They can climb smooth lianes, and fearlessly forage at all heights through the smallest branches of forest canopies. They range widely in search of rich but temporary and patchy resources like birds’ nests, insects, seeds and fruit [95].

Pregnant or lactating females are trapped only in the austral spring and summer of normal years, between mid September and mid-April, occasionally to midwinter of a good fruiting season. The normal seasonal breeding of ship rats produces annual changes in abundance, from low numbers in spring and early summer to an autumn peak [64,96]. After a beech masting event, rats bred all year round [97]. Measured density in mainland forests ranges from 1–12/ha [98]. Abundance indices for ship rats derived from trap catch or footprint tracking rates predictably surge after heavy seed masting of beech [99,100] or rimu trees [101]. However, the precise mechanisms behind these irruptions are not well known.

### 6.3. Predators and Competitors

Ship rats are a staple dietary item for feral cats and stoats, less often for ferrets and weasels. Native predators are too scarce to trouble them.

In theory, no predators can eat rats faster than they can be replaced [49] or prevent a rat irruption if food supplies favour it, because the intrinsic rate of increase of rodents is much higher than that of any carnivore [102]. However, predators can potentially delay the start of an irruption, hasten its decline, or limit rat population recovery if food is later scarce and the natural mortality rate of rats is high [51]. Trapping stoats and cats for kiwi protection in Northland is often followed by increased captures of rats [103]. Sustained trapping of stoats in beech forests increases rat tracking rates [93].

At the broadest landscape scale, ship rat populations range from generally scarce but irruptive in cooler, beech-dominated forests, to common in warmer, non-beech forests. If true, this suggests that average ship rat abundance will increase as the New Zealand climate warms [104].

Ship rats in New Zealand dominate the other three rodent species, via strong interspecific competition and the differential effects of predation. They replace the last few Pacific rats wherever (rarely) the two meet, and outcompete the larger Norway rat for food in forests, [68] confining Norways to foraging on the ground in commensal and riparian habitats [69]. Ship rats readily hunt and eat mice [46], and in forests the distributions of ship rats and mice are strongly reciprocal. Australian brushtail possums compete with ship rats for seeds and fruit, but are larger and more aggressive, so removal of possums is predictably followed by increased numbers of ship rats [48].

Although New Zealand’s native wildlife had already been damaged for 600 years by Pacific rats, and for 150 years by Norway rats, the arrival of ship rats in the mid to late nineteenth century posed additional, new threats [105]. All three rat species have been responsible in their turn for the loss of many large, flightless, ground-dwelling endemic invertebrates, such as giant weevils and giant wētā, that are now either extinct or confined to rodent-free islands [106]. In beech forests, foraging by ship rats (and mice) on litter-dwelling invertebrates has altered the below-ground community of decomposers [107]. The carnage following the arrival of ship rats on Big South Cape Island in 1964 was responsible for the loss of five of the six endemic bird taxa resident on the island, but it also stimulated official recognition of the need to manage rat invasions on islands [108].

The ship rat is the only one of the three rat species still common, so remains the primary target of contemporary conservation rescue work. Ship rats are by far the widest-distributed and most abundant predators contributing to the local and/or total limitation of twelve of 30 New Zealand forest bird species [105]. Unfortunately, the wider damage due to ship rats alone cannot now be separated from the effects of massive habitat destruction and the spread of mustelids. Ship rats as prey also indirectly affect important endemic species. For example, after rats are removed by a successful control operation, hungry stoats may turn to prey more on birds [109]. The ship rat is one of seven key taxa selected for eradication under the Predator-Free New Zealand programme [110], but this is not financially or socially feasible with current tools.

## 7. Feral Ferret, *Mustela furo* (Linnaeus, 1758)

The ferret is a domesticated form of the European polecat, *M. p. putorius*, with which it can still interbreed [111], but there is no trace of polecat ancestry in the New Zealand feral population. Ferrets have the typical mustelid long, narrow body and short legs, and weigh on average around 1.3 kg males, 0.6 kg females.

### 7.1. Arrival

New Zealand’s political identity started in 1840 as a colony of the British Empire. In the early 19th century, its economy depended largely on exports of wool, its primary product. Wild European rabbits were introduced into New Zealand for meat and sport in the 1850s, and by the mid-1870s, huge populations of rabbits were causing enormous and unexpected damage to sheep pastures and to the wool industry. Pastoral farmers assumed that rabbit numbers were normally kept down by their ‘natural enemies’, but the rabbits had been introduced without them. The runholders pressed the colonial government to introduce rabbit predators too, starting with ferrets [73].

Domesticated ferrets are generally docile and amenable to handle, and easy to breed in captivity. Teams of ferrets and dogs had traditionally been employed by gamekeepers for rabbit control in UK, and were already working in Australia. Because ferrets were readily obtainable, and were seen as natural rabbit-killing specialists, they were the first participants in what became the earliest known attempt to use vertebrate predators to achieve biological control of a vertebrate pest [112].

Ferrets were imported in several large consignments from Europe in the 1870s, but their mortality in the early shipments (due to canine distemper or inexpert handling on board), was very high. In 1882 the New Zealand government discontinued the importations, and established domestic ferret breeding studs instead. Over the next few decades, at least 75,000 ferrets were distributed around the South Island’s rabbit-infested sheep pastures, and others in the North Island. Many cage-bred youngsters did not adapt well to life in the wild, but eventually the survivors established largest known population of truly feral ferrets in the world. They not only failed to control the rabbits, but were themselves soon added to the list of unwelcome introduced pests. Their legal protection (as officially designated rabbit predators) gave way to control campaigns starting in the 1930s [113].

Ferrets live mainly in open country, avoiding forests throughout the two main islands. They do not swim well, and have not been carried to any other islands. Trapped feral ferrets are still generally much easier to handle than stoats, which were never domesticated.

### 7.2. Abundance

The distribution and abundance of ferrets depends mainly on those of rabbits, via the primary effect of rabbit numbers on the productivity of female ferrets. Ferrets generally mate once a year, conceiving 9–10 embryos in spring (September and October). The embryos are implanted without delay, and are gestated for 41–42 days. The actual number of kits born in October or November is fewer (usually 4–8), depending on the nutritional condition of the female. Lactating mothers can be found until December [114]. Later conceptions or second litters in one season are rare and seldom successful.

Juvenile ferrets of both sexes emerge from natal dens in early summer, and when c. 3 months old they disperse up to >45 km in search of their own home range [115]. Up to 50% of newborn ferrets die before they reach trappable age. Recruitment rates can be up to c. 3/km^2^ in January–February, inversely correlated with adult density [116] and food supply [117]. Expectation of further life for newly independent juveniles is ~1.3 years [118], higher after resident adults have been culled, improving after the first year of age but decreasing by 50% a year after age 5 years.

Ferret density in good rabbit habitat can reach up 10.1 ferrets per km^2^ depending on the time of year, rabbit abundance, and elevation, or down to <2.9 ferrets per km^2^ in semi-arid habitats [116]. Abundance surveys covering large areas (>20 km^2^) usually find the highest numbers in late summer (March–April), but have to be adjusted for seasonal differences in ferret behaviour, especially trap avoidance in winter [119]. When rabbit numbers fall, so does juvenile recruitment, and ferret abundance follows [120]. So, contrary to the original reason for importing them, ferrets have no effect on the numbers or distribution of rabbits; rather, rabbits determine ferret numbers [87].

### 7.3. Predators and Competitors

Adult ferrets have few predators in New Zealand except humans, and their only effective competitors are feral cats. Ferrets dominate stoats, but coexist with them at landscape scale by mutual avoidance [121]. In open country, ferrets far outnumber stoats [122]. except after ferret removal, more likely due to competitive exclusion than habitat choice.

The effects of human predation on feral ferrets in New Zealand is relatively well known, because ferrets carry and spread bovine tuberculosis (bTB), a serious threat to New Zealand’s agricultural industry unless long-term herd prevalence in cattle is maintained to below 0.2%. Hence, agricultural authorities have strongly invested in research on the significance of ferrets to the management of this important disease [123]. Infectious ferrets not only amplify bTB, they also freely disperse it across the landscape, and can transmit to domestic stock [124], starting new outbreaks of bTB in areas previously free of it [124]. Ferrets are also acutely vulnerable to canine distemper virus, and can easily catch it from farm dogs.

Ferrets are significant to conservation of native species because they are (with dogs) the main predators of adult kiwi, which can otherwise be long-lived [125]. In the Tongariro Forest Kiwi Sanctuary, five-yearly applications of aerial 1080 protect radio-tagged adult kiwi and increase the kiwi population for the first two years of each cycle, but both measures reverse as ferrets come back [126]. Northland was once a stronghold of kiwi populations, but the arrival of ferrets there in the late 1970s precipitated a steep decline in the abundance and range of kiwi [127]. A successful rabbit control operation or an outbreak of the rabbit disease RHD prompts ferrets to switch to native prey such as banded dotterel nests [128].

## 8. Stoat, *Mustela erminea* L.

The stoat is a small but hardy predator native to the northern hemisphere. Stoats range from Siberia, where they hunt the fluctuating numbers of lemmings and voles under snow, to Britain, where they are rabbit specialists. Stoats have a long thin body, short legs, and a long black-tipped tail. They are agile hunters, capable of foraging in the canopy as easily as any squirrel, or underground in rabbit burrows. Body size varies considerably in different habitats of New Zealand, but males are consistently much larger, averaging around 320 g, c.50% heavier than females that average c. 215g.

### 8.1. Arrival

The stoat is one of the last two deliberately introduced ‘natural enemies of the rabbit’, brought to New Zealand in the late nineteenth century. When domesticated ferrets proved not hardy enough to control the vast numbers of rabbits in the cold South Island high country, the colonial government was pressed by desperate wool barons to introduce stoats and weasels instead. The first trial shipment of 10 was successfully imported into Dunedin in March 1883, stimulating an official importation programme that lasted until 1892 [129]. Over that decade, at least 25 shipments arrived, from which at least 7838 stoats and weasels (often not distinguished) were landed, and released on the worst-infested pastures teeming with rabbits [130]. Within six years there were reports of stoats spreading into the forests far from any known releases, including to places where there were as yet no rabbits [61].

Stoats may now be found throughout the two main and on many inshore islands. They were never deliberately taken to any islands further offshore, so they are still absent from Stewart, Great Barrier, Little Barrier and the Chatham Is. However, stoats swim readily and well, potentially up to c. 5 km of the mainland coast [131]. Any island of high value for wildlife within stoat swimming distance is in perpetual danger of stoat invasion.

### 8.2. Abundance

Stoats are flexible and opportunist in their diets, and can live in any habitat in which they can find prey, at any elevation from sea shore to alpine zone. They always have to be on the alert for attack from larger predators, so tend to avoid open country unless they can keep under cover. Stoats are much more common in forests than are ferrets, and vice versa in grassland [122]. In general, stoat numbers vary more, and can reach much higher peaks, in beech forest than in podocarp–broadleaf forest [132].

Stoats eat almost any small animals they can catch, preferably rats, mice, rabbits, possums, birds and, when more substantial prey are scarce, many native invertebrates [133,134]. When rats or mice are abundant, stoats concentrate on them, but a decline in abundance of rodents causes a rapid shift to alternative resources, often detectable as a strong and consistent functional response by stoats to eat more birds [135]. Bird eggs are a favourite food; on the wide braided bed of the Tasman River, 32% (30/94) of stoat dens and 26% (23/88) of scats collected in spring contained eggshell [136].

In the breeding season in late winter/early summer (September to November) male stoats actively search for receptive females, but there is no pair bond, and no paternal care. Fertilized ova develop for ~2 weeks, then the blastocysts go into a prolonged period of delay. The number of corpora lutea, visible as yellow dots on the ovaries over the next 9–10 months, is a direct measure of a high (10–13) ovulation rate [137,138]. Stimulated by increasing daylight hours in the following spring, the blastocysts implant and the embryos develop to full term in ~4 weeks. Juvenile females are extraordinarily precocious, and are sexually mature as small nestlings. In early summer they are fertilized in the den by the same male that serves their mother [138], and then, like her, enter a compulsory period of delayed implantation when the blastocysts float free in the uterus until the following spring.

Delayed implantation means that fertility (litter size) cannot be related to fecundity (ovulation rate), which is fixed at mating, but over the winter months before the next birthing season, litter size is finely adjusted to the prevailing food supplies. Resorption and nestling mortality cut down the potential litter size by 0–100% of ova fertilized, depending on the female’s nutritional condition during the season of implantation and lactation [137,138]. Therefore, maximum potential fecundity is set during the mating season of one year, while productivity cannot be realised (in litters of usually 8–10) until the following year.

Vertebrate food resources for stoats in New Zealand forests are of two major types, representing the opposite ends of a spectrum of rodent dynamics [104]. (1) Beech forests covering nearly 3 million ha of mainly mountain land offer fluctuating feast-or-famine supplies of mice (±rats) comparable with the ‘cycles’ of lemmings and voles in the boreal environment in which stoats evolved [42]. (2) Podocarp/mixed forests, often at lower elevations, support widespread and more reliable populations of ship rats (but fewer mice) [92].

Stoat population dynamics in the two main forest types are strongly influenced by their different patterns of rodent abundance. The three-stage correlation between masting events and the abundances of rodents and stoats is well established and mostly quite predictable [139]. In beech forests after a masting event, mouse numbers rapidly increase over winter, and by spring the well fed overwintered female stoats of all ages can minimize the intrauterine mortality of their litters [140]. In the early summer (8–9 months after the fall), huge numbers of newly independent young stoats appear [99]. Their numbers are due not to additional productivity of female stoats (as in weasels) but to a great improvement in juvenile survival of the litters conceived the previous year [138]. Post-seedfall stoat irruptions can reach five-to-six times their normal summer abundance over very short periods. A similar sequence of events can be observed after a good fruiting season in podocarp forests [141].

By contrast, in non-mast years and poor habitats, scarcer supplies of rodents cause female stoats to resorb some of their embryos, or even all of them, and others bear their young but fail to rear them. Stoat productivity drops, at worst even down to total reproductive failure [43].

The population dynamics of stoats through a typical cycle of pulsed resources are controlled by age- and sex-specific variations in fertility and survival. The full potential productivity of female stoats of all ages is reached only in the areas or years with the highest density of small mammal prey, even though very few of the young born then live long enough to breed in their turn. That means that realistic population modelling of stoats is impossible without age-, year- and sex-specific survival data [142].

First year mortality rates (sexes pooled) among South Island beech forest stoats varied from 0.55 to 0.92/year, reducing to 0.30–0.75/year in adults 1–5 years old. Oddly, survival to 3 years old among adults born when mice were abundant was lower than in those born when mice were scarce [143]. In mixed podocarp and exotic forests in the North Island, mortality rate estimated over 5 years’ sampling averaged 0.76 [144].

There is no agreement as to whether stoat predation is strong enough to affect numbers of introduced mammals [49]. The disappearance of Pacific and Norway rats from most of the South Island at about the time that stoats arrived cannot be distinguished from competition between rat species. Stoats cannot prevent irruptions of mice [51], which inevitably decline after young mice cease to join the population, whether stoats are present or not [41]. Field observations may [93] or may not [48] support expectations of mesopredator release of rats or rabbits after removal of stoats.

Post-masting stoat irruptions usually cause seriously increased mortality among threatened hole-nesting species such as kākā and mohua [145]. The damage is most severe *at the time* rodents are abundant, not (as in the more complex animal communities of the northern hemisphere) *after* they decline, because alternative foods in beech forests are scarce, and higher numbers of stoats each taking about the same number of birds as usual adds up to greater losses for birds. The associated mouse irruptions are seldom high enough to divert the stoats’ attention from eating birds [146]. However, this sequence of events is predictable enough to enable conservation authorities to plan some six months ahead for effective control operations against stoats and rats together, with demonstrable benefits for forest birds [147]. In non-beech habitats stoat abundance is generally lower and more stable, but stoats still threaten the survival of kiwi chicks, most years, even though these may form only a small part of their diet [148].

### 8.3. Predators and Competitors

Stoats cannot compete for rabbits against cats or ferrets, so are dominated by both. Captive stoats avoid foraging too close to a caged cat or ferret [121], but these avoid forests, which are good hunting grounds for stoats. Elsewhere, stoats can co-exist with them either by hunting at different times of day, or by taking over areas where ferrets and cats have been removed. On the other hand, stoats can easily dominate weasels, which tend to be rare or absent where stoats are common [149].

Stoats are liable to be attacked by falcons (*Falco novaeseelandiae*), or weka (*Gallirallus a. australis*), but the general numbers of stoats are not affected by natural predators or accidental deaths.

The main predators of stoats in New Zealand, as elsewhere, are humans. Stoats are widely blamed for both the historic extinctions of endemic species and for the low density of surviving native populations, but in fact they arrived too late, or never met, many extinct populations, whereas these losses are much more often due to rats. Research investment into finding ways to reduce the abundance of stoats often emphasises the serious ecological mistake made by the early European colonists in bringing them to New Zealand, although the previous damage by rats is usually underestimated or ignored.

## 9. Weasel, *Mustela nivalis vulgaris* Erxleben, 1777

### 9.1. Arrival

The weasel was introduced to New Zealand at the same time as the stoat, and for the same reasons. It is similar in colour and general appearance to the stoat, but much smaller, with a short tail lacking a black tip. Despite their smaller size, weasels run and climb as well as stoats. Males usually weigh up to c. 150 g, about twice as large as females.

Both weasels and stoats were easily captured by English gamekeepers collecting stock for transportation, but weasels were more common, so many more weasels than stoats were shipped to New Zealand (incomplete records mention at least 2622 weasels to 963 stoats). A total of 25 known shipments, carrying at least 7838 weasels and stoats (the two were distinguished in only 15 of the 25 cargo lists) arrived over the decade after 1883 [129]. Contemporary reports document the spread of both species across the country [130].

### 9.2. Abundance

Weasels were at first hugely abundant. Guthrie-Smith [150] described weasels as arriving in waves at Tūtira in 1902 (*before* the rabbits!) but quickly disappearing, so by the time rabbits reached the district, the tide of weasels had passed on. Thomson [61] quotes a report of a large and rapid but short-lived irruption of weasels on the Westland coast in 1889. (Both reports assume the authors could distinguish weasels from stoats). However, by 1950, weasels had become ‘exceedingly rare’ everywhere [22]. Early predator control programs reported catching vastly fewer weasels than stoats. Weasels remain patchily distributed over most of the two main islands. They are known to have reached only one offshore island, Maud, by unknown means.

The reproductive cycle of the weasel is quite different from that of stoats. Weasels have a much longer fertile season (September to March) and no delay in implantation, so the ovaries contain corpora lutea only when the female is actively pregnant. Average fecundity (ovulation rate 7.1), embryo count (5.7); birth rate (6.2), litter size (4.5) [151] are all lower than those of stoats [152], but weasels can be much quicker to respond to a sudden increase in food supply [153]. When adult females are very well fed (but not otherwise) they can produce a second litter, and young females born early in spring can breed in mid summer, although they seldom do so in the wild. So, unlike stoats, the weasels’ freedom from delayed implantation enables them to produce more than one litter a year, allowing a much faster population response when prey increase, especially if stoats decrease at the same time. Hence, the numbers and distributions of local populations of weasels are never stable, ranging from occasional irruptions to frequent local extinctions, correlated with the fluctuating distribution and abundance of small prey (mostly mice, lizards, birds, eggs and invertebrates).

Weasels in New Zealand are greatly disadvantaged by the absence of their primary prey, the various species of voles (Family Cricetidae). Weasels have survived in New Zealand by responding to every local aggregation of small prey, however temporary. One irruption of mice in a young pine plantation at Pureora Forest Park was observed to stimulate a short-lived aggregation of weasels under the protection of the thick sward of ungrazed grass [144]. Weasels are able to follow mice into their burrows and nests, and can extract lizards from small refuges. However, in general they are scarce enough to cause minimal concern relative to the other introduced predators.

### 9.3. Predators and Competitors

Weasels in New Zealand are vulnerable to occasional attack by all larger predators, especially cats, stoats, ferrets and harriers, but these do not control the numbers of weasels. Interference competition is a more frequent danger. Effective, long term removal of stoats for biodiversity protection is increasingly benefitting weasels. Reports of increasing abundance of weasels are emerging in places where stoat numbers have been reduced, in, e.g., kiwi sanctuaries and similar protected areas after years of stoat trapping [130]. More weasels tend to appear after aerial 1080 operations in the South Island, because mice can detect and avoid 1080, so such operations remove rats and stoats but often leave mice and weasels to increase.

The remarkable decline in abundance of weasels relative to that of stoats since their first introduction together might be explained by (1) historic changes in the abundance of prey small enough for weasels, or (2) competition (both exploitative and interference) between weasels and stoats. Or, maybe both in turn.

First, the first weasels invading the 19th century landscape of New Zealand could still find plenty of native substitutes for the missing voles, such as ground-nesting birds, bats, lizards, frogs, wētā and other large invertebrates as well as house mice and nestling rats [53]. So long as these remained abundant and widespread on the mainland, weasels could increase in numbers much more rapidly than stoats. The weasels’ capacity for a very high population rate of increase could well explain their huge initial abundance, not only because more weasels were released, but also because well-fed weasels could out-breed stoats so long as small prey remained so plentiful. Now, that advantage has been lost.

Second, weasels and stoats in their native countries both depend on the same prey resources, but in their native patchy and variable environments, they can co-exist under a shifting balance of advantages. Weasels depending on voles move rapidly between patches, and avoid confrontations with stoats, while the stoats’ wider diet enables them to avoid depending on a single prey resource [154]. Weasels can quickly respond to increased numbers of voles, but are vulnerable to local extinction when voles decline. Stoats can range widely for voles and larger prey such as rabbits, but are limited by delayed implantation to producing only one litter a year. Stoats dominate weasels when they meet, but weasels can escape into vole burrows too small for stoats [154]. In New Zealand, the absence of voles, the weasel’s key resource, makes the weasel’s vole-specialist strategy much less effective than the stoat’s ability to tackle a wider range of prey. Over time, these differences have tilted the long-term balance in relative abundance strongly in favour of stoats.

## 10. Feral Cat, *Felis catus* Linnaeus, 1758

### 10.1. Arrival

The ships of early European traders and visitors were always infested with rats and/or mice, so also carried cats supposed to control them. Cook noticed one of the *Resolution*’s cats hunting ashore in Dusky Sound, in March 1773. As European and American shipping increased, cats were landed, left behind or donated to Māori at intervals [155,156,157], or were adopted by settlers, but truly independent feral cats did not appear in the bush until after the late 1820s [158]. Māori valued cats for their meat and fur, and by the 1840s, cats were common in Māori villages [159], but ‘kept tied up like dogs’ [160].

### 10.2. Abundance

Feral cats become established in parts of the North Island by the late 1830s, and by 1840 feral cats were being blamed for the ‘destruction of many indigenous species’ [21]. They were present in Canterbury by 1850, and ‘very numerous’ there by 1860 [161]. They were carried inland with miners and settlers plagued by rodents, so were soon distributed throughout all three main islands, both in cities and as fully or intermittently feral. In the 1880s, when plagues of rabbits were ruining pastoral leaseholds, farmers fetched cats from the cities to release on rabbit-infested farmland [162]. They were often carried around the archipelago on the ships of sealers and whalers, and lighthouse keepers, so are, or have been, present on at least 33 islands of >5 ha, including large, biologically important reserves.

In forest of the Orongorongo Valley, changes in numbers of mice and rats counted in cat scats matched changes in the numbers of mice and rats trapped [163]. When the numbers of cats declined, rat abundance increased to levels five times that previously observed [164]. Rabbits are the preferred food of cats, and in open country and during seasons when young rabbits are easily available, cat guts contain many rabbit remains [165].The breeding success of cats is strongly influenced by the spring/summer abundance of rabbits [166], leading to variation in the measured density of feral cats on farmland from zero to high, 3–5.6/km^2^ [167]. On Herekopare Island, which supported a large breeding population of seabirds, the density of feral cats was 1.18/ha just before they were eradicated [168].

The arrival of the rabbit disease RHD combined with cat predation had a profoundly depressing effect on the population dynamics of the remaining rabbits [87]. On 11% of 80 islands (generally smaller ones), cats introduced as a biological control agent succeeded in eradicating rabbits [169]. Conversely, removal of cats (and mustelids) can allow rabbits to recover [170], but in general, cats do not much affect rabbit numbers.

Feral cats are or have been of serious conservation concern on offshore islands of high wildlife value, because they have always been more likely than any mustelids to be carried around on ships, but they have since died out on some islands and been eradicated from others. The eradication of cats from Little Barrier Island in 1980 [171] backfired when it permitted more intense predation by Pacific rats on Cook’s petrel nests, which was reversed only after the rats were also eradicated in 2004 [172].

Feral cats also threaten many species of native ground nesting shorebirds, wetland birds, lizards and forest birds [173,174], but are much more difficult to control than other predators. Lizard populations declined rapidly on the mainland after cats were introduced, some to extinction, and still do whenever cats (and ferrets) are suddenly deprived of rabbits [86].

### 10.3. Predators and Competitors

Cats have no effective natural enemies in New Zealand, except humans, especially conservation agencies working to protect threatened native species and game birds, and they are the top competitor in the New Zealand carnivore guild.

## 11. European Hedgehog, *Erinaceus europaeus occidentalis* Barrett-Hamilton, 1900

### 11.1. Arrival

Hedgehogs were the last of the European small mammal species deliberately introduced into New Zealand, and the only insectivores. Bodyweights in New Zealand average 620–700 g for most of the year, falling to 540–650 g in winter. The exact origins of the New Zealand stock is unknown [175]. They were brought to remind homesick settlers of their native countries, and because they were believed to be effective natural enemies of garden pests. Many hedgehogs were imported between 1907 and 1912 to control slugs and snails, although there is no evidence that they do.

### 11.2. Abundance

Hedgehogs adapted easily to New Zealand’s mild climate and rich grasslands full of invertebrates, so soon became enormously abundant. North Island dairy country is ideal habitat for hedgehogs, so they are still very common there, and in suburban gardens that offer plenty of food and dry den sites. Hedgehogs are fewer on cold South Island braided riverbeds and in wet rainforests, but can live in exotic pine forests and any grassy habitats where slugs, snails, beetles, and worms are most easily available. They are present on five of the larger offshore islands, but scarce in wet (<2500 mm rain a year) and frosty (>250 frost days a year) areas.

An average hedgehog eats up to around 160 g a day, comprising the great majority of the biomass of invertebrates (averaging 740 g/ha/night) estimated to be taken by introduced mammals from the forest floor [105]. Hedgehogs also kill any small ground vertebrates they find (mice, lizards, frogs, birds, skinks), destroy many broods of eggs, and scavenge carrion from traps.

Hedgehogs are most active after dusk, and quickly learn to navigate to find temporary food sources such as bird nests with eggs. They construct their dens on dry, well-drained sites, for use as daytime retreats or for winter hibernation [176,177]. They can travel long distances; 16 of 65 marked hedgehogs in the Mackenzie Basin travelled more than 5 km, up to 12 km, over two years, often using up all 900 m of thread available in a spool backpack in one night [178].

Hedgehogs save energy with short periods of daily torpor, and in cooler regions begin to hibernate when the mean earth temperature drops below 10–11 °C. The length of the hibernation period varies from short or zero in frost-free parts of Northland to up to 3 months in the South Island, but even in colder climates some will awake every few days, so be vulnerable to trapping in winter [92].

Relative trapping indices confirm that hedgehogs can be very common in New Zealand. After a decline in rabbits due to RHD in the upper Waitaki Basin, 101,650 trapnights set at 14 trap sites caught 1067 hedgehogs [122]. At another site at Macraes Flat, North Otago, >2000 hedgehogs were removed over 3 years from only 2100 ha [179]. Hence, hedgehogs join the list of introduced predators threatening native fauna, especially lizards and ground-nesting birds.

However, hedgehogs in New Zealand are now apparently less abundant than they once were. Hedgehogs are often killed on the road, and repeated counts can act as a useful proxy for landscape-scale population surveys, varying with season and habitat. In the 1950s, the numbers of road-killed hedgehogs recorded in New Zealand (in places, 25–50/100 km or more) outnumbered those on British roads 30–40-fold, but have now fallen in both countries (North Island, 1.7/100 km in 2005; UK 1.5/100 km in 2004). The latest South Island count (12.5/100 km in 2007) was ~12 times higher than on any North Island road since 1990 [180]. These estimates might appear to infer real changes in hedgehog densities, but they do not take into account other influential factors such as variations in traffic density and habitats adjacent to the roads surveyed.

### 11.3. Predators and Competitors

Hedgehogs compete with native insectivores for any available prey on the ground, and can deplete a large proportion of the total stock of forest invertebrates available to important native species such as the kiwi. They compete with other predators for eggs of ground-nesting birds. However, they have no effective predators except humans.

## 12. Australian Brushtail Possum, *Trichosurus vulpecula* (Kerr, 1792)

The brushtail possum is one of 13 species of Australian marsupials introduced to New Zealand, but the only one to establish a permanent population. Eight species of wallabies, imported for sport hunting, plus four other marsupial species, did not survive. The brushtail possum has a rich pelt very attractive to dealers in luxurious fur, and none of the protections that inhibit harvesting of other fur-bearing mammals available in other parts of the world. Its size and weight vary greatly around the country, averaging 2–3 kg, increasing southwards. There are two general colour forms, grey and black, each with much variation. The marsupial pouch has two teats. The skull is heavy, with a fenestrated palate. Possums are largely arboreal, but commonly interact with the other small mammals both in the canopy and on the ground.

### 12.1. Arrival

Possums were introduced by settlers in the hope of establishing a profitable industry from otherwise unproductive forests. The first successful release, in 1858 near Riverton, Southland, was followed by larger-scale importations and redistributions organized by regional acclimatization societies mostly between 1890 and 1900. Only about 200–300 individuals were imported, from which the consequent spread of possums was accelerated by additional releases of the New Zealand-bred progeny of the original introductions.

From 1922 onwards, increasing evidence of the detrimental effects of possums on native forests and orchards led to disputes between people holding differing attitudes to possums, as pests to be managed versus as a resource to be protected, generating contradictory changes in legislation. Illegal releases were particularly common from 1920 to 1940 [181]. In 1947-47, all protection was withdrawn, and the deliberate introduction of possums was recognized as a major mistake. Nevertheless, possums colonised the two main islands plus 19 offshore and outlying islands. Established populations have subsequently been eradicated from 14 offshore and outlying islands of a wide range of sizes (1–2321 ha).

### 12.2. Abundance

Invasion by possums into a new areas invariably starts with a slow but steady increase in numbers, then a relatively short-lived irruption to a peak population causing severe damage to vegetation, and finally a drastic decline to more stable postpeak levels, averaging less than half the former peak numbers. As abundance declines, possums become less fecund, less fat, lighter and older on average, and breed later [182], although these effects can be partially reversed if food supplies improve [183]. This historic pattern has been well documented in the Taramakau Valley, in Westland and in South Westland. In normal years, numbers are usually highest in February–May as the seasonal influx of newly independent young arrives, and lowest after winter mortality in September–October has taken its toll.

In general, abundance of possums varies with forest type and elevation, ranging from 1–3/ha in pine forest, 1–9/ha in farmland and 10–12 possums/ha in mixed podocarp–broadleaf forest, to >15.0/ha at forest margins within 250 m of pasture, where the diversity and biomass of understorey species is greatest, particularly if livestock are excluded. [184]. In 2009, the total number of possums in New Zealand was estimated at ~30 million [185], and the main biotic and abiotic factors influencing possum abundance have recently been identified [186].

Annual monitoring of possum populations has been done over many years in the Pararaki catchment in southern Wairarapa, and in the Orongorongo Valley near Wellington. Analysis of population trends at Pararaki suggests an ‘irruptive fluctuation’ pattern, explaining the coupled dynamics of possums and their food supply [187]. By contrast, possums in the Orongorongo area show little or no long-term trend in abundance, only a short-term (4-year) cycle fluctuating between 6.5 and 13.7/ha (mean 9.8) observed over the period 1967 and 1998. The Orongorongo Valley possum population illustrates a stationary population regulated over a long-term (>10-year) timescale by a delayed density dependent link between reproductive success and food supply [188]. It probably represents the natural behaviour of long-established possum populations in unmanaged areas, as a modest annual fluctuation around the mean.

In good habitat, the mortality of pouch young between birth and independence ranges from <20 to >70%, mostly at ~3–4 months of age. Few independent young survive to 2 years of age [189]. Of 116 pouch young born in Orongorongo Valley in 1979 and 1980, 39% died in the pouch and 38% disappeared (and almost certainly died) between pouch emergence and 9 months of age; of the remaining 23%, 37% died on or near their natal area and 22% dispersed; only 11 survived from first pouch emergence at 4–5 months to 2 years of age on their natal area [190]. In forest a female, on average, weans 1.5 young by 3 years of age; in pasture/scrub or colonising populations 2.0 young, or more [191]. Annual adult natural mortalities average 15–30%, generally lowest at 2–4 years, then increasing with age, but the further life expectancy of 3–4-year-old possums is still ~5 years [192].

The dynamics of possum populations are of intense and well-funded interest to wildlife managers in New Zealand, and have been modelled in relation to browsing damage [193], controlled harvesting of fur for maximum sustainable yield [194], and bTB control [195]. For example, monitoring a recovering population after experimental removal of possums from two 6-ha areas of forest found an increase in the percentage of females breeding from c. 80% to 100%, and improved survival of pouch young to weaning. Possum abundance had been reduced to near-zero density, but after 2 years it recovered to 32–56% of its former numbers, and the exponential rate of population growth during the next 2.5 years was 0.59 [183]. The maximum population growth rate of 0.77 per year suggests that >54% of individuals would have to be removed each year to eliminate a population [102].

### 12.3. Predators and Competitors

Feral cats are the second most frequent predators of possums, after humans. Up to 38% of cat scats collected in the Orongorongo Valley contained possum hair or bones [163]. Young possums may be taken occasionally by Australasian harriers/kahu (*Circus approximans*). Possums are frequently run over by vehicles, with unknown consequences for local populations [180], but natural predation is probably much less significant to possum abundance than is human activity.

Possum skins were in great demand from fur traders in 1978–82, when >2 million skins/year were exported, enough to reduce possum abundance in accessible areas, and the average size of skins offered for sale declined. The anti-fur movement overseas, aimed to protect rare fur-bearing species, collapsed the New Zealand market for pelts in the late 1980s, even though possums are among the few fur-bearers that need no such protection. Annual possum harvest fell to c. 250,000/year in 1996–98 [196]. Since 2000, the development of mixed possum fur/wool fibres has renewed interest in possums as a commercial product. On average 18 possums yield 1 kg of loose fur, worth > NZ$100 per kg, which has stimulated renewed harvesting of wild possums. In 2014, possum-related garments fetched between $100 and $150 million per year from a harvest of ~2 million possums [197].

Possums are the principal wildlife maintenance host for bovine TB in New Zealand, and their transmission of the disease to livestock is a matter of major economic significance [198]. Typically, 1–10%, up to 60%, of possums in a TB-infected population show macroscopic lesions. Most infected possums can continue to live and shed the disease around their environment for 3–6 months [199,200].

Because brushtail possums in New Zealand have few parasites and natural predators, and they can browse on forage containing fewer toxic compounds than those in Australia, they can reach two- to 20-fold higher numbers in New Zealand, compared with mainland Australia, where their density is usually less than 1/ha. Their much higher abundance in New Zealand, plus the vulnerability of New Zealand plants and fauna that have evolved in the absence of browsing mammals, make possums among the worst introduced conservation pests. Possums compete for nest sites with hole-nesting birds, such as kiwis, parakeets, and saddlebacks [201], and for food with ship rats. Landscape-scale aerial 1080 poisoning operations can remove possums, with significant benefits for common forest birds [126], except that removing possums also benefits ship rats. Possums may also prey directly on native bats, and may compete with them for food [202]. Populations of native snails are severely damaged by possums; a single possum is capable of eating more than 60 *Powelliphanta* snails in a night.

Commercial trappers kill many possums each year, and when the price of fur is high, trapping may affect possum numbers significantly [203]. Since 2014, large areas of New Zealand have been subject to control of possums, rats and stoats to prevent irruptions of pest populations during years following a heavy beech seedfall [147]. Community groups also tirelessly patrol trapping grids to help control possums and other pests for the benefit of native fauna in many areas of New Zealand [204].

Possums have been eradicated from more than 20 fenced sanctuaries in New Zealand [205]. The technology required to eradicate them from large unfenced mainland areas is advancing; for example, the Cape to City program aims to control possums and other pests over 26,000 ha of rural and peri-urban land in Hawke’s Bay [206]. The Predator-Free New Zealand initiative aims for national eradication of possums, rats and stoats by the year 2050 [110], and is driving significant improvement in possum control technologies [207].

## 13. Conclusions

The history and ecology of the small mammals of New Zealand provides paradigm examples of two of the most widespread general observations in ecology. (1) The abundance of small, short-lived species is determined primarily by their seasonal breeding success, which in turn depends entirely on habitat quality and food supplies. (2) The population dynamics of small mammal predators responds to the abundance and distribution of prey, not vice versa.

The eleven species described here live in an environment with a relatively low mammalian diversity by northern standards, so their interactions with each other (competitive and predatory) can often be more easy to see. They include seven species of primary consumers (rats, mice, rabbits, possums and hedgehogs), which on first arrival have all in turn staged massive irruptions in numbers; and four secondary consumers (mustelids and cats), which, contrary to expectations, have been unable to prevent or control such irruptions—rather, they have accompanied the changes in the abundance of their prey as ‘passengers rather than drivers’. To understand why, this account gives equal attention to both.

All eleven species are opportunists, descended from ancestors that had evolved among competitive continental faunas, which were then granted an unusual opportunity to invade a remote archipelago of islands that never had an indigenous fauna of small mammals. All soon occupied new environments packed with suitable food supplies capable of supporting a huge initial abundance. Three of them (*Rattus exulans* and *Mustela nivalis* everywhere, and *R. novergicus* outside urban areas) are now scarcer than before, though not yet extinct. The other eight survive in the wild in unstable numbers controlled by their variable food resources, which predictably determine their annual fluctuations in breeding success. Their continued patchy but widespread distribution is typical of opportunists whose populations are primarily controlled from the bottom up, tempered by their interactions with each other.

As many of the above species accounts can confirm, competition within the small mammal community is often important even when it can be observed only indirectly. Mice clearly avoid rats, rats avoid possums, weasels avoid stoats, stoats avoid cats. A classic case of mesopredator release of Pacific rats was observed when cats were removed from Little Barrier Island, and other examples can confidently be expected inside any fenced sanctuary whenever removal of ship rats releases the breeding potential of mice.

All species except cats and hedgehogs are subject to predation by other members of the small mammal community, but the most significant direct losses for all of them are usually due to food shortage or intermittent human control programmes. For both predators and their prey, the success or otherwise of the most recent breeding season is the best predictor of distributions and subsequent population numbers, via food-related controls on annual birth and death rates. In the ‘breed fast, die young’ life history strategy of all eleven species, fertility and mortality rates are variable but usually high at all ages, most critically at the time of juvenile recruitment. All eleven species are or have been regarded as pests threatening New Zealand’s indigenous fauna, especially the most valuable remaining endemic species, hence attracting lethal attention from human conservationists.

Research funding is always scarce, and usually linked to some national imperative. Successive New Zealand governments have invested huge resources into research on the population dynamics of rabbits as economic pests of agriculture, possums and ferrets as carriers of bovine TB, and mice/rats/stoats through the beechmast cycle as a recurring threat to native biodiversity. To design rational control strategies for these species, the detailed, age-specific mechanisms controlling their natural variation in fertility and mortality rates must be known. This requires a much greater effort than is usually available for monitoring projects limited to counting carcasses, which explains why these details are not known for all the species reviewed here. Now, the greatest injection of research funding ever is being provided under the current Predator-Free New Zealand programme, expected to achieve unprecedented results by 2015 [110]. Who knows what major advances might be possible by then!

## Figures and Tables

**Table 1:** Sequence of first arrival dates of small mammal species colonising the New Zealand archipelago.

Date (Years AD)	Species	Reasons for Introduction, and Route
c. 1280	*Rattus exulans*	Accidental, from Polynesian canoes
After 1769	*Mus musculus, R. norvegicus, Felis catus*	Accidental, from European and American ships
1850s	*Oryctolagus cuniculus*	Deliberate, by settlers for meat and sport
1860s	*R. rattus*	Accidental, from European and American ships
1870s	*Mustela furo*	Deliberate, by pastoralists for rabbit control
1880s	*M. erminea, M. nivalis*	Deliberate, by pastoralists for rabbit control
1890s	*Erinaceus europaeus occidentalis*	Deliberate, by settlers to control garden pests
1890s	*Trichosurus vulpecula*	Deliberate, by settlers to establish a fur industry

## Data Availability

This paper summaries published information into an original connected narrative. The primary data can be found in the references cited.
